# The Role of Nutrition in Pediatric Gastrointestinal Diseases

**DOI:** 10.3390/nu18081307

**Published:** 2026-04-21

**Authors:** Ron Shaoul, Andrew S. Day

**Affiliations:** 1 Pediatric Gastroenterology & Nutrition Institute, Ruth Children’s Hospital of Haifa, Rambam Medical Center, Faculty of Medicine, Technion, Israel Institute of Technology, Haifa 31096, Israel; 2 Department of Paediatrics and Child Health, University of Otago Christchurch, Christchurch 8140, New Zealand; andrew.day@otago.ac.nz

Nutrition has both a supportive and therapeutic role in the management of many gastrointestinal (GI) diseases [1,2]. Optimized nutrition is essential for the growth and development of all children [3]. This is especially relevant to children with GI conditions that can variably affect nutrient intake, absorption, or metabolism.

There is no other time in life where the provision of adequate and balanced nutrition is as crucial than during infancy and childhood [4,5]. During this dynamic phase characterized by rapid growth, development, and developmental plasticity, a sufficient amount and appropriate composition of nutrients both in health and disease are of key importance for growth, functional outcomes such as cognition and immune response, and the metabolic programming of long-term health and well-beings.

The main role of the gut is to digest and absorb nutrients in order to maintain life and well-being. Chronic conditions affecting the GI tract commonly impact nutrition adversely. This is especially relevant in children and adolescents with chronic GI conditions, where growth and development are key outcomes. The nutritional impacts of chronic GI conditions in childhood include weight loss or reduced weight gain, impaired linear growth, and delayed pubertal development [2,6,7]. In addition, the malabsorption or reduced intake of micronutrients may lead to specific deficiencies, with further potential adverse effects.

Conditions that can have an impact on nutrition include inflammatory bowel disease (IBD), eosinophilic disorders, celiac disease, cystic fibrosis or other pancreatic disorders, chronic liver disease, intestinal failure, neurological disorders, functional GI disorders, cancer, and obesity. This Special Issue aimed to focus on the nutritional aspects of various pediatric GI conditions.

Magen Rimon et al. [8] reviewed the evidence for dietary interventions in IBD (Crohn’s disease [CD], ulcerative colitis [UC]) and their effects on symptoms and intestinal inflammation. Diet is an important environmental factor in IBD pathogenesis. Many diets improve symptoms (including overlapping functional GI symptoms), but few have strong proven anti-inflammatory effects. Most studies are small, often in mild–moderate disease, limiting generalizability. The introduction of exclusive enteral nutrition (EEN) as a treatment option for induction of remission in CD was a breakthrough in disease pathophysiology understanding and has paved the way for dietary options based on this understanding. Six to eight weeks of EEN is still considered the first-line for induction of remission in children with active CD; it has been shown effective in adults but is less commonly used. EEN shows comparable clinical remission to corticosteroids in children but shows superior mucosal healing. The proposed mechanisms include: exclusion of food antigens, nutritional correction, microbiome shifts (reduced diversity), barrier improvement and direct anti-inflammatory effects. EEN may help in complicated CD and show promise in acute severe UC in small trials.

The Crohn’s Disease Exclusion Diet (CDED) was shown to be as effective as EEN for induction in children and better tolerated. CDED was also shown to be effective in some adults. CD-TREAT and “Tasty and Healthy” aim to mimic the effects of EEN with whole foods; early data are encouraging but need replication. Partial Enteral Nutrition (PEN) (formula + regular food) is generally less effective than EEN for induction, but evidence supports PEN for maintenance of remission as an adjunct.

The Ulcerative Colitis Exclusion Diet (UCED) is a phased exclusion diet tested in small cohorts (children and adults with refractory disease); some clinical responses have been observed but data are limited. Other less proven diets include: Specific Carbohydrate Diet (SCD), Low-FODMAP diet, IgG4-guided exclusion diet Anti-Inflammatory Diet (AID). Another promising diet is the Mediterranean diet: one RCT found similar remission rates to SCD but easier adherence and broader health benefits.

Spector Cohen et al. [9] reviewed the nutritional strategies for intestinal rehabilitation in children with Short Bowel Syndrome (SBS). Management is based on three stages: acute (0–weeks), adaptation (months–1–2 yrs) and maintenance (years). Anatomy (residual small bowel length, presence of ileocecal valve, colon continuity) strongly predicts outcomes and tailoring of nutrition.

During the Acute phase the key steps are to start parenteral nutrition (PN) early to maintain hydration/nutrition and to initiate enteral nutrition (EN) as soon as clinically feasible to stimulate adaptation. While oral or gastric delivery of EN is preferred, post-pyloric feeding may be required in some children. The type and pattern of enteral feeding needs to be tolerance-based; continuous feeding improves absorption/tolerance in many cases but bolus mimics physiology. Furthermore, breast milk is preferred for infants; donor milk and fortified strategies should be considered when maternal milk unavailable.

The goals of the Adaptation phase are to gradually advance EN and proportionally reduce PN guided by stool/stoma output, growth, and electrolyte levels. Diet composition should be individualized by anatomy: this includes considering the balance between long and medium chain triglycerides, limited simple sugars, and consideration of soluble fiber if the colon present and monitoring oxalate risk with ileal loss. Fluid and electrolyte management (e.g., use of oral rehydration solution as required, urinary sodium monitoring) is essential.

In the third, Maintenance, phase the key goals are to aim for enteral autonomy (EA) with multidisciplinary support. This includes stepwise PN weaning and pharmacologic adjuncts (e.g., teduglutide) as needed. Close monitor for adverse outcomes such as oral aversion important, with provision of feeding skill therapy as required. Long-term surveillance for micronutrient deficiencies, metabolic bone disease, hydration and growth is mandatory.

This report highlighted numerous evidence gaps. Most recommendations derive from observational studies and expert consensus; randomized clinical trials in children with SBS are scarce. Substantial practice variability exists. The authors called for prospective multicenter trials, biomarkers to predict EA, and research on diet–microbiome interactions and whole-food/blenderized feeds.

The authors concluded that enteral nutrition is the central, modifiable driver of intestinal adaptation in children with SBS. The optimal timing, feeding modality and composition must be individualized according to residual anatomy and phase of rehabilitation. Finally, current practice relies largely on observational evidence, so multicenter prospective studies are urgently needed to define best practices.

Papoutsaki et al. [10] completed a systematic review of observational studies to assess short-term (≥6 to <12 months) and long-term (≥12 months) effects of a gluten-free diet (GFD) on nutrient intake, blood micronutrient status, anthropometry, body composition and dietary habits in children/adolescents (0–18 y) with celiac disease (CD). Fifteen studies were included with 2004 participants (1053 with CD, 951 controls without CD). The 15 studies had various designs (cross-sectional, case–control and prospective). Diet was assessed by different methods (e.g., food frequency questionnaires (FFQ), 24-h recalls and food diaries).

The authors found that GFD improves intestinal healing but many nutrient intakes remain inadequate or become imbalanced (notably vitamin D, iron, calcium, folate, magnesium, some B-vitamins and zinc). Nutritional problems are often present at diagnosis and many persist despite GFD; deficiencies may reflect overall diet quality and frequent use of processed gluten-free products. Routine, early and ongoing nutritional assessment (including labs and anthropometry), dietitian-led education, targeted supplementation when needed, and regular follow-up (first recheck ~6 months, then annually) are recommended. The authors call for higher-quality, standardized prospective studies to inform pediatric guidance.

Sarbagili-Shabat et al. [11] undertook a systematic review looking at growth, nutritional status, and body composition in pediatric-onset UC. Fifteen studies were included with 1575 participants: five studies were prospective, five cross-sectional and five retrospective.

The prevalence of growth impairment/growth failure varied widely (7–36%) depending on definitions and timing. Weight deficits were more commonly reported than height deficits. Final adult height was generally comparable to controls overall, but males diagnosed before puberty showed an increased risk for reduced adult height in some cohorts.

Undernutrition prevalence ranged up to ~25% in some cohorts; in contrast, overweight/obesity were also observed in multiple studies. BMI z-scores were sometimes lower vs. controls despite low stunting rates, indicating early weight depletion relative to height.

Very limited data was available for body composition: (five small studies with between 12 and 40 participants). Findings were inconsistent: two studies reported reduced lean mass/phase angle in UC vs. controls, others found no significant differences. Longitudinal data suggest lean mass can improve with disease control. Assessment methods were mainly bioelectrical impedance (BIA) and skinfold measurements. None of the reports included dual energy absorptiometry (DEXA) studies.

The authors summarized that children and adolescents with UC show a variable but real signal of compromised nutritional status and growth—most often weight depletion and sometimes reduced lean mass—yet evidence is limited, heterogeneous, and underpowered. Llarger, focused studies using standardized definitions and accurate body-composition methods (e.g., DEXA) are needed to define prevalence, drivers, and optimal monitoring/intervention strategies.

Saito et al. [12] completed a multi-center retrospective observational study across six Japanese children’s hospitals (patients 0–18 years) assessing in-hospital oral sodium selenite preparations: usage, serum concentrations, dosing, and safety (AEs). They included 472 eligible children after exclusions; primary analyses used 239 “selenium-treated” cases (with serum selenium data) and 220 matched “selenium-untreated” reference cases for AE comparisons. Median age 1.3 years, gastrointestinal disorders ~46% and congenital conditions ~36% were most common.

The authors concluded that hospital-compounded oral sodium selenite, given at ~2–3 µg/kg/day and monitored, achieves expected serum selenium levels and—within the observed dosing range below published upper tolerable limits—was not associated with an increased rate of adverse events in predominantly children with GI conditions. However, prospective, controlled studies and standardized monitoring are still needed.

Together: these five reports provide important perspectives about the vitally important intersections between nutrition and GI disorders in children. Further studies remain important to provide further information to guide management options and to enhance outcomes.
